# 8-Iodo­quinolinium triiodide tetra­hydro­furan solvate

**DOI:** 10.1107/S1600536808031516

**Published:** 2008-10-04

**Authors:** Jung-Ho Son, James D. Hoefelmeyer

**Affiliations:** a Department of Chemistry, The University of South Dakota, 414 E. Clark Street, Vermillion, SD 57069, USA

## Abstract

The title compound, C_9_H_7_IN^+^·I_3_^−^·C_4_H_8_O, was synthesized from 8-amino­quinoline using the Sandmeyer reaction. The 8-iodo­quinolinium cation is essentially planar and the triiodide ion is almost linear. N—H⋯O hydrogen bonds, and inter­molecular I⋯I [3.7100 (5) Å] and I⋯H inter­actions, between the cation, anion and solvent mol­ecules result in the formation of sheets oriented parallel to the (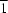
03) plane. Between the sheets, 8-iodo­quinolinium and triiodide ions are stacked alternately, with I⋯C distances in the range ∼3.8–4.0 Å.

## Related literature

For the synthesis, see: Lucas & Kennedy (1943 ▶); Sandmeyer (1884 ▶). For related structures, see: Son & Hoefelmeyer (2008 ▶); Svensson & Kloo (2003 ▶).
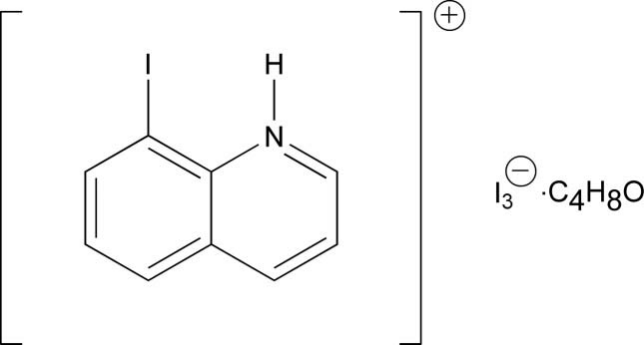


## Experimental

### 

#### Crystal data

C_9_H_7_IN^+^·I_3_^−^·C_4_H_8_O
*M* *_r_* = 708.86
Monoclinic, 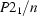

*a* = 7.8674 (4) Å
*b* = 17.6510 (9) Å
*c* = 13.1465 (7) Å
β = 90.343 (1)°
*V* = 1825.59 (16) Å^3^
*Z* = 4
Mo *K*α radiation
μ = 6.82 mm^−1^
*T* = 100 (2) K
0.57 × 0.36 × 0.27 mm

#### Data collection

Bruker SMART APEXII diffractometer
Absorption correction: numerical (*XPREP* in *SHELXTL*; Sheldrick, 2008 ▶), *T*_min_ = 0.111, *T*_max_ = 0.262
18253 measured reflections
3363 independent reflections
3291 reflections with *I* > 2σ(*I*)
*R*_int_ = 0.079

#### Refinement

*R*[*F* ^2^ > 2σ(*F* ^2^)] = 0.026
*wR*(*F* ^2^) = 0.111
*S* = 0.97
3363 reflections
172 parameters
H-atom parameters constrained
Δρ_max_ = 0.55 e Å^−3^
Δρ_min_ = −2.63 e Å^−3^

### 

Data collection: *APEX2* (Bruker, 2006 ▶); cell refinement: *SAINT* (Bruker, 2006 ▶); data reduction: *SAINT*; program(s) used to solve structure: *SHELXTL* (Sheldrick, 2008 ▶); program(s) used to refine structure: *SHELXTL*; molecular graphics: *ORTEP-3* (Farrugia, 1997 ▶) and *Mercury* (Macrae *et al.*, 2006 ▶); software used to prepare material for publication: *SHELXTL*.

## Supplementary Material

Crystal structure: contains datablocks I, global. DOI: 10.1107/S1600536808031516/ci2653sup1.cif

Structure factors: contains datablocks I. DOI: 10.1107/S1600536808031516/ci2653Isup2.hkl

Additional supplementary materials:  crystallographic information; 3D view; checkCIF report

## Figures and Tables

**Table 1:** Hydrogen-bond geometry (Å, °)

*D*—H⋯*A*	*D*—H	H⋯*A*	*D*⋯*A*	*D*—H⋯*A*
N1—H1⋯I1	0.88	2.80	3.297 (4)	117
N1—H1⋯O1^i^	0.88	1.94	2.690 (5)	142

Symmetry code: (i) 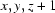.

**Fig. 1.**
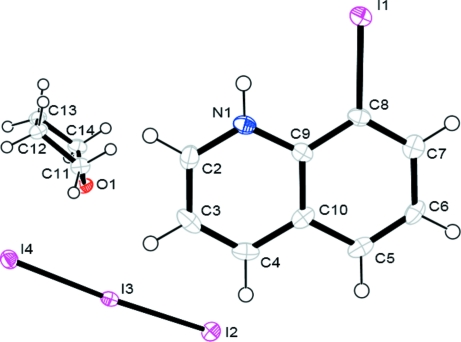
Asymmetric unit of 8-iodoquinolinium triiodide.THF. Displacement ellipsoids are drawn at the 50% probability level.

**Fig. 2.**
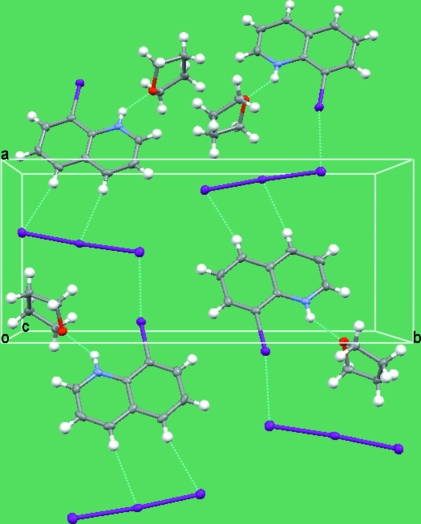
Crystal structure of 8-iodoquinolinium triiodide.THF, viewed along the c axis. Dotted lines denote hydrogen bonding and close contacts. Displacement ellipsoids are drawn at the 50% probability level.

## supplementary crystallographic information

### Comment

8-Iodoquinoline is a starting material for the synthesis of 8-substituted quinoline derivatives. The molecule 8-iodoquinoline was synthesized starting from 8-aminoquinoline using the Sandmeyer reaction (Sandmeyer, 1884), similar to the synthesis of iodobenzene (Lucas & Kennedy, 1943). During its synthesis, two 8-iodoquinolinium salt crystals, 8-iodoquinolinium chloride dihydrate (Son & Hoefelmeyer, 2008) and 8-iodoquinolinium triiodide.THF were isolated. The synthesis, characterization and crystal structure of 8-iodoquinolinium triiodide^.^THF are reported here.

The 8-iodoquinolinium cation is essentially planar (Fig. 1). The C9—C8—I1 angle is 121.9 (3) °, slightly larger than the ideal value of 120°. A weak intermolecular interaction is present between atom I1 of the cation and I2 of the triiodide anion; I1···I2(1 + *x*, *y*, 1 + *z*) = 3.7100 (5) Å. The geometry of triiodide is almost linear, with I2—I3 = 2.9416 (8) Å, I3—I4 = 2.9014 (8) Å and I4—I3—I2 angle is 177.445 (17)°. The shape resembles symmetric, free triiodide that typically occurs in the presence of bulky cations (Svensson & Kloo, 2003).

The 8-iodoquinolinium cation, I_3_^-^ and THF form an extended sheet (Fig. 2) parallel to the (1 0 3) plane through I1···I2, I3···H4 (3.15 Å) and I4···H5 (3.09 Å) interactions and N—H···O hydrogen bonds (Table 1). Between the sheets, 8-iodoquinolinium and I_3_^-^ ions are stacked alternately, with the I2···C2^ii^, I3···C8^ii^ and I3···C9^ii^ distances being 3.742 (5), 3.840 (4) and 3.838 (4) Å, respectively [symmetry code: (ii) -1/2 + *x*, 1/2 - *y*, -1/2 + *z*].

### Experimental

In a 500 ml beaker, a mixture of 8-aminoquinoline (10 g, 0.069 mol) and water (50 ml) was heated with stirring. While cooling the mixture in an ice bath, concentrated HCl (50 ml) was added to form a red solution. NaNO_2_ (7.8 g, 0.113 mol) was dissolved in water (50 ml) and cooled in an ice bath separately. NaNO_2_ solution was slowly transferred to the 8-aminoquinoline solution. Light brown precipitate formed during the addition step but eventually disappeared to form a reddish transparent solution. KI (17.9 g, 0.108 mol) was dissolved in water (25 ml) and added to the reaction mixture. Bubbles and brownish vapor evolved during addition. The solution turned to dark brown with black precipitate. The solution was refluxed with a watch glass on top of the beaker, and it became reddish brown with formation of a heavy organic layer; the black precipitate remained. After cooling and standing overnight, golden brown crystals of 8-iodoquinolinium chloride dihydrate had formed spontaneously in the solution. The mixture was neutralized upon addition of NaOH solution, which led to dissolution of the golden brown crystals and retention of the black precipitate. The liquid portion was separated from the black precipitate. 8-Iodoquinoline could be recoverd from the liquid portion upon extraction with toluene. The black precipitate was dissolved in THF and crystallized by pentane vapor diffusion to obtain the crystal of 8-iodoquinolinium triiodide^.^THF [yield: 7.14 g (15%), m.p. 366–368 K]. ^1^H NMR (acetone-*d*): 7.647–7.725 (dd, 1H, quin C*H*), 8.237–8.305 (dd, 1H, quin C*H*), 8.339–8.385 (dd, 1H, quin C*H*), 8.559–8.602 (dd, 1H, quin C*H*), 9.260–9.309 (dd, 1H, quin C*H*), 9.380–9.415 (dd, 1H, quin C*H*), 12.999 (br, 1H, N*H*). ^13^C NMR (acetone-*d*): 91.517 (quin *C*8), 124.089 (quin *C*H), 130.558 (quin *C*9/10), 130.817 (quin *C*H), 132.183 (quin *C*H), 138.064 (quin *C*9/10), 146.463 (quin *C*H), 147.614 (quin *C*H), 149.842 (quin *C*H). Elemental analysis result suggests that about 75% of THF depleted during storage. Analysis calculated for C_13_H_15_I_4_NO: C 22.03, H 2.13, N 1.98%; found: C 19.30, H 1.32, N 2.19%.

### Refinement

H atoms were positioned geometrically (N—H = 0.88 Å and C—H = 0.93 Å) and allowed to ride on the parent atoms with *U*_iso_(H) = 1.2*U*_eq_(C).

### Crystal data

**Table:**

C_9_H_7_IN^+^·I_3_^−^·C_4_H_8_O	*F*(000) = 1280
*M**_r_* = 708.86	*D*_x_ = 2.579 Mg m^−^^3^
Monoclinic, *P*2_1_/*n*	Melting point: 367 K
Hall symbol: -P 2yn	Mo *K*α radiation, λ = 0.71073 Å
*a* = 7.8674 (4) Å	Cell parameters from 9984 reflections
*b* = 17.6510 (9) Å	θ = 2.8–25.4°
*c* = 13.1465 (7) Å	µ = 6.82 mm^−^^1^
β = 90.343 (1)°	*T* = 100 K
*V* = 1825.59 (16) Å^3^	Block, metallic violet
*Z* = 4	0.57 × 0.36 × 0.27 mm

### Data collection

**Table:**

Bruker SMART APEXII diffractometer	3363 independent reflections
Radiation source: fine-focus sealed tube	3291 reflections with *I* > 2σ(*I*)
graphite	*R*_int_ = 0.079
ω scans	θ_max_ = 25.4°, θ_min_ = 1.9°
Absorption correction: numerical (XPREP in SHELXTL; Sheldrick, 2008) or (SADABS; Bruker, 2003)???	*h* = −9→9
*T*_min_ = 0.111, *T*_max_ = 0.262	*k* = −21→21
18253 measured reflections	*l* = −15→15

### Refinement

**Table:**

Refinement on *F*^2^	Primary atom site location: structure-invariant direct methods
Least-squares matrix: full	Secondary atom site location: difference Fourier map
*R*[*F*^2^ > 2σ(*F*^2^)] = 0.027	Hydrogen site location: inferred from neighbouring sites
*wR*(*F*^2^) = 0.111	H-atom parameters constrained
*S* = 0.98	*w* = 1/[σ^2^(*F*_o_^2^) + (0.0437*P*)^2^ + 2.836*P*] where *P* = (*F*_o_^2^ + 2*F*_c_^2^)/3
3363 reflections	(Δ/σ)_max_ = 0.001
172 parameters	Δρ_max_ = 0.55 e Å^−^^3^
0 restraints	Δρ_min_ = −2.63 e Å^−^^3^

### Special details

**Table:**

Geometry. All e.s.d.'s (except the e.s.d. in the dihedral angle between two l.s. planes) are estimated using the full covariance matrix. The cell e.s.d.'s are taken into account individually in the estimation of e.s.d.'s in distances, angles and torsion angles; correlations between e.s.d.'s in cell parameters are only used when they are defined by crystal symmetry. An approximate (isotropic) treatment of cell e.s.d.'s is used for estimating e.s.d.'s involving l.s. planes.
Refinement. Refinement of *F*^2^ against ALL reflections. The weighted *R*-factor *wR* and goodness of fit *S* are based on *F*^2^, conventional *R*-factors *R* are based on *F*, with *F* set to zero for negative *F*^2^. The threshold expression of *F*^2^ > σ(*F*^2^) is used only for calculating *R*-factors(gt) *etc*. and is not relevant to the choice of reflections for refinement. *R*-factors based on *F*^2^ are statistically about twice as large as those based on *F*, and *R*- factors based on ALL data will be even larger.

### Fractional atomic coordinates and isotropic or equivalent isotropic displacement parameters (Å^2^)

**Table:**

	*x*	*y*	*z*	*U*_iso_*/*U*_eq_	
C2	0.2020 (7)	0.3301 (2)	0.9044 (4)	0.0231 (10)	
H2	0.2425	0.3807	0.9094	0.028*	
C3	0.0503 (6)	0.3161 (3)	0.8562 (4)	0.0265 (10)	
H3	−0.0138	0.3565	0.8275	0.032*	
C4	−0.0077 (5)	0.2427 (3)	0.8500 (3)	0.0229 (9)	
H4	−0.1134	0.2322	0.8177	0.027*	
C5	0.0356 (6)	0.1062 (3)	0.8839 (3)	0.0234 (9)	
H5	−0.0706	0.0943	0.8531	0.028*	
C6	0.1379 (6)	0.0497 (3)	0.9214 (3)	0.0231 (9)	
H6	0.1035	−0.0017	0.9155	0.028*	
C7	0.2952 (6)	0.0678 (3)	0.9690 (3)	0.0221 (9)	
H7	0.3640	0.0281	0.9954	0.027*	
C8	0.3493 (5)	0.1402 (3)	0.9775 (3)	0.0181 (8)	
C9	0.2463 (5)	0.1992 (3)	0.9385 (3)	0.0167 (8)	
C10	0.0900 (6)	0.1829 (3)	0.8917 (3)	0.0197 (9)	
C11	0.5434 (6)	0.3891 (3)	0.1252 (3)	0.0233 (9)	
H11A	0.6024	0.3486	0.1637	0.028*	
H11B	0.4297	0.3972	0.1551	0.028*	
C12	0.6463 (6)	0.4619 (3)	0.1272 (4)	0.0250 (9)	
H12A	0.7212	0.4638	0.1879	0.030*	
H12B	0.5708	0.5068	0.1275	0.030*	
C13	0.7520 (6)	0.4587 (3)	0.0285 (3)	0.0243 (9)	
H13A	0.7269	0.5028	−0.0158	0.029*	
H13B	0.8753	0.4578	0.0439	0.029*	
C14	0.6948 (5)	0.3849 (3)	−0.0216 (3)	0.0237 (9)	
H14A	0.6885	0.3907	−0.0965	0.028*	
H14B	0.7749	0.3433	−0.0052	0.028*	
I1	0.58420 (4)	0.162595 (15)	1.04875 (2)	0.02127 (14)	
I2	−0.00026 (4)	0.166821 (16)	0.18354 (2)	0.02404 (14)	
I3	0.05172 (4)	0.330240 (15)	0.21544 (2)	0.01836 (14)	
I4	0.11961 (4)	0.488485 (17)	0.25933 (2)	0.02294 (14)	
N1	0.2941 (4)	0.2734 (2)	0.9445 (3)	0.0182 (7)	
H1	0.3897	0.2845	0.9762	0.022*	
O1	0.5292 (4)	0.36930 (18)	0.0195 (2)	0.0210 (6)	

### Atomic displacement parameters (Å^2^)

**Table:**

	*U*^11^	*U*^22^	*U*^33^	*U*^12^	*U*^13^	*U*^23^
C2	0.026 (3)	0.017 (2)	0.027 (2)	−0.0006 (16)	0.003 (2)	0.0016 (16)
C3	0.022 (2)	0.034 (3)	0.023 (2)	0.012 (2)	0.0013 (18)	0.005 (2)
C4	0.015 (2)	0.035 (3)	0.018 (2)	−0.0014 (18)	−0.0022 (15)	−0.0022 (18)
C5	0.022 (2)	0.029 (2)	0.020 (2)	−0.0101 (19)	0.0027 (16)	−0.0036 (18)
C6	0.030 (2)	0.017 (2)	0.022 (2)	−0.0091 (18)	0.0006 (17)	−0.0029 (17)
C7	0.025 (2)	0.021 (2)	0.020 (2)	0.0005 (17)	0.0047 (17)	−0.0024 (17)
C8	0.0144 (19)	0.025 (2)	0.0151 (19)	0.0012 (18)	0.0005 (15)	−0.0010 (17)
C9	0.0149 (19)	0.022 (2)	0.0128 (17)	0.0022 (17)	0.0027 (14)	0.0011 (16)
C10	0.022 (2)	0.022 (2)	0.014 (2)	−0.0064 (17)	0.0038 (16)	−0.0040 (17)
C11	0.022 (2)	0.029 (2)	0.019 (2)	−0.0016 (18)	0.0018 (16)	−0.0011 (18)
C12	0.022 (2)	0.023 (2)	0.030 (2)	−0.0029 (18)	−0.0053 (18)	−0.0038 (19)
C13	0.020 (2)	0.026 (2)	0.027 (2)	−0.0063 (19)	−0.0055 (17)	0.0035 (19)
C14	0.017 (2)	0.032 (3)	0.022 (2)	−0.0001 (18)	0.0041 (17)	0.0014 (18)
I1	0.0187 (2)	0.0219 (2)	0.0232 (2)	0.00115 (10)	−0.00448 (14)	0.00111 (10)
I2	0.0264 (2)	0.0248 (2)	0.0209 (2)	−0.00137 (10)	0.00150 (15)	−0.00241 (10)
I3	0.0143 (2)	0.0245 (2)	0.0163 (2)	0.00172 (9)	−0.00029 (13)	0.00112 (9)
I4	0.0240 (2)	0.0216 (2)	0.0232 (2)	0.00220 (10)	−0.00198 (14)	0.00159 (10)
N1	0.0137 (17)	0.024 (2)	0.0172 (17)	0.0021 (14)	0.0010 (13)	−0.0015 (14)
O1	0.0182 (15)	0.0214 (16)	0.0233 (15)	−0.0030 (12)	0.0010 (12)	−0.0034 (13)

### Geometric parameters (Å, °)

**Table:**

C2—N1	1.343 (6)	C9—C10	1.401 (7)
C2—C3	1.370 (8)	C11—O1	1.436 (5)
C2—H2	0.95	C11—C12	1.519 (6)
C3—C4	1.375 (7)	C11—H11A	0.99
C3—H3	0.95	C11—H11B	0.99
C4—C10	1.415 (7)	C12—C13	1.547 (6)
C4—H4	0.95	C12—H12A	0.99
C5—C6	1.372 (7)	C12—H12B	0.99
C5—C10	1.423 (6)	C13—C14	1.527 (6)
C5—H5	0.95	C13—H13A	0.99
C6—C7	1.419 (7)	C13—H13B	0.99
C6—H6	0.95	C14—O1	1.440 (5)
C7—C8	1.352 (6)	C14—H14A	0.99
C7—H7	0.95	C14—H14B	0.99
C8—C9	1.413 (6)	I2—I3	2.9430 (4)
C8—I1	2.104 (4)	I3—I4	2.9011 (4)
C9—N1	1.364 (6)	N1—H1	0.88
			
N1—C2—C3	120.9 (4)	O1—C11—H11A	110.7
N1—C2—H2	119.5	C12—C11—H11A	110.7
C3—C2—H2	119.5	O1—C11—H11B	110.7
C2—C3—C4	119.0 (4)	C12—C11—H11B	110.7
C2—C3—H3	120.5	H11A—C11—H11B	108.8
C4—C3—H3	120.5	C11—C12—C13	104.1 (4)
C3—C4—C10	120.0 (4)	C11—C12—H12A	110.9
C3—C4—H4	120.0	C13—C12—H12A	110.9
C10—C4—H4	120.0	C11—C12—H12B	110.9
C6—C5—C10	119.4 (4)	C13—C12—H12B	110.9
C6—C5—H5	120.3	H12A—C12—H12B	109.0
C10—C5—H5	120.3	C14—C13—C12	103.6 (3)
C5—C6—C7	120.1 (4)	C14—C13—H13A	111.0
C5—C6—H6	119.9	C12—C13—H13A	111.0
C7—C6—H6	119.9	C14—C13—H13B	111.0
C8—C7—C6	121.6 (4)	C12—C13—H13B	111.0
C8—C7—H7	119.2	H13A—C13—H13B	109.0
C6—C7—H7	119.2	O1—C14—C13	105.4 (3)
C7—C8—C9	119.1 (4)	O1—C14—H14A	110.7
C7—C8—I1	119.3 (3)	C13—C14—H14A	110.7
C9—C8—I1	121.6 (3)	O1—C14—H14B	110.7
N1—C9—C10	117.6 (4)	C13—C14—H14B	110.7
N1—C9—C8	121.9 (4)	H14A—C14—H14B	108.8
C10—C9—C8	120.5 (4)	I4—I3—I2	175.753 (14)
C9—C10—C4	119.4 (4)	C2—N1—C9	123.0 (4)
C9—C10—C5	119.3 (5)	C2—N1—H1	118.5
C4—C10—C5	121.3 (4)	C9—N1—H1	118.5
O1—C11—C12	105.2 (3)	C11—O1—C14	104.5 (3)
			
N1—C2—C3—C4	0.5 (7)	C8—C9—C10—C5	0.1 (6)
C2—C3—C4—C10	−0.9 (7)	C3—C4—C10—C9	−0.4 (7)
C10—C5—C6—C7	1.1 (7)	C3—C4—C10—C5	−178.2 (4)
C5—C6—C7—C8	−0.9 (7)	C6—C5—C10—C9	−0.7 (6)
C6—C7—C8—C9	0.3 (6)	C6—C5—C10—C4	177.1 (4)
C6—C7—C8—I1	−179.9 (3)	O1—C11—C12—C13	−25.5 (4)
C7—C8—C9—N1	−179.8 (4)	C11—C12—C13—C14	1.4 (5)
I1—C8—C9—N1	0.4 (5)	C12—C13—C14—O1	23.0 (4)
C7—C8—C9—C10	0.0 (6)	C3—C2—N1—C9	1.4 (7)
I1—C8—C9—C10	−179.8 (3)	C10—C9—N1—C2	−2.7 (6)
N1—C9—C10—C4	2.2 (6)	C8—C9—N1—C2	177.1 (4)
C8—C9—C10—C4	−177.7 (4)	C12—C11—O1—C14	41.1 (4)
N1—C9—C10—C5	−180.0 (4)	C13—C14—O1—C11	−40.2 (4)

### Hydrogen-bond geometry (Å, °)

**Table:**

*D*—H···*A*	*D*—H	H···*A*	*D*···*A*	*D*—H···*A*
N1—H1···I1	0.88	2.80	3.297 (4)	117
N1—H1···O1^i^	0.88	1.94	2.690 (5)	142

Symmetry codes: (i) *x*, *y*, *z*+1.
